# A Case of Unwitnessed Ingestion: Highlighting the Diagnostic Challenge in High-Risk Patients

**DOI:** 10.7759/cureus.96362

**Published:** 2025-11-08

**Authors:** Adrian N Burman

**Affiliations:** 1 Emergency Medicine, Northwick Park Hospital, London, GBR

**Keywords:** button battery ingestion, education and communication, high-risk group, paediatric patient, pediatrics emergency, swallowed foreign body

## Abstract

In the paediatric emergency medicine setting, the ingestion of foreign bodies is an important topic due to its ability to rapidly progress to a life-threatening emergency and the often difficult history-taking of the paediatric population. It is also a relatively common presentation, largely managed conservatively; however, it can also necessitate immediate invasive intervention. In this case, we explore the management of a three-year-old boy who presented with black stool but otherwise a normal assessment, who was subsequently found to have ingested a 20 mm button battery requiring emergency endoscopic removal. The main lesson from this case is to have a high index of suspicion for ingested foreign bodies in children with communication difficulties and a history of non-food item ingestion; this applies to both clinicians and parents.

## Introduction

The ingestion of button batteries has emerged as a critical paediatric emergency over the past four decades, with the number of reported cases in the United States of America (USA) increasing by more than 4.6 times [1]. Ingestion of button batteries primarily affects children under six years of age, who accounted for 66% of US button battery ingestions in 1985-2019. This age group is also at a higher risk of complications following ingestion [1-3].

Whilst many foreign objects pass through the digestive tract without complication, those that become lodged in the oesophagus can cause significant morbidity and mortality within hours through localised tissue injury. As battery size increases, particularly >20 mm, they may cause caustic injury within 30 minutes, with a subsequent risk of perforation or fistula formation [2].

The European Society for Paediatric Gastroenterology, Hepatology and Nutrition (ESPGHAN) released a position paper summarising the evidence and providing guidelines for the management of ingested button batteries. It also highlights the difficult nature of diagnosis in unwitnessed ingestions, as symptoms may be more vague and may only present once complications have developed. These symptoms include haematemesis, haemoptysis, melaena, abdominal pain, weight loss, chest pain, cough, stridor, hoarseness, sore throat, fever, neck stiffness, or even acute haemorrhage [4]. This highlights the need for a high index of suspicion for foreign body/button battery ingestion in patients at risk of ingestion.

This case specifically focuses on the need for a high index of suspicion for button battery ingestion for both clinicians and parents in the context of particularly high-risk children and the difficulty in assessing such patients.

## Case presentation

The patient in question was a three-year-old boy awaiting assessment for autism and known to suffer from chronic constipation. He was regularly taking multiple herbal vitamins and probiotic supplements due to a limited diet (including Nutrigen Vegy Syrup, Bioray Kids Probiotic, and NDF Focus Bioray Kids). Developmentally, he was minimally verbal and followed only a few commands, which posed difficulties with history taking and a thorough examination.

**Figure 1:**
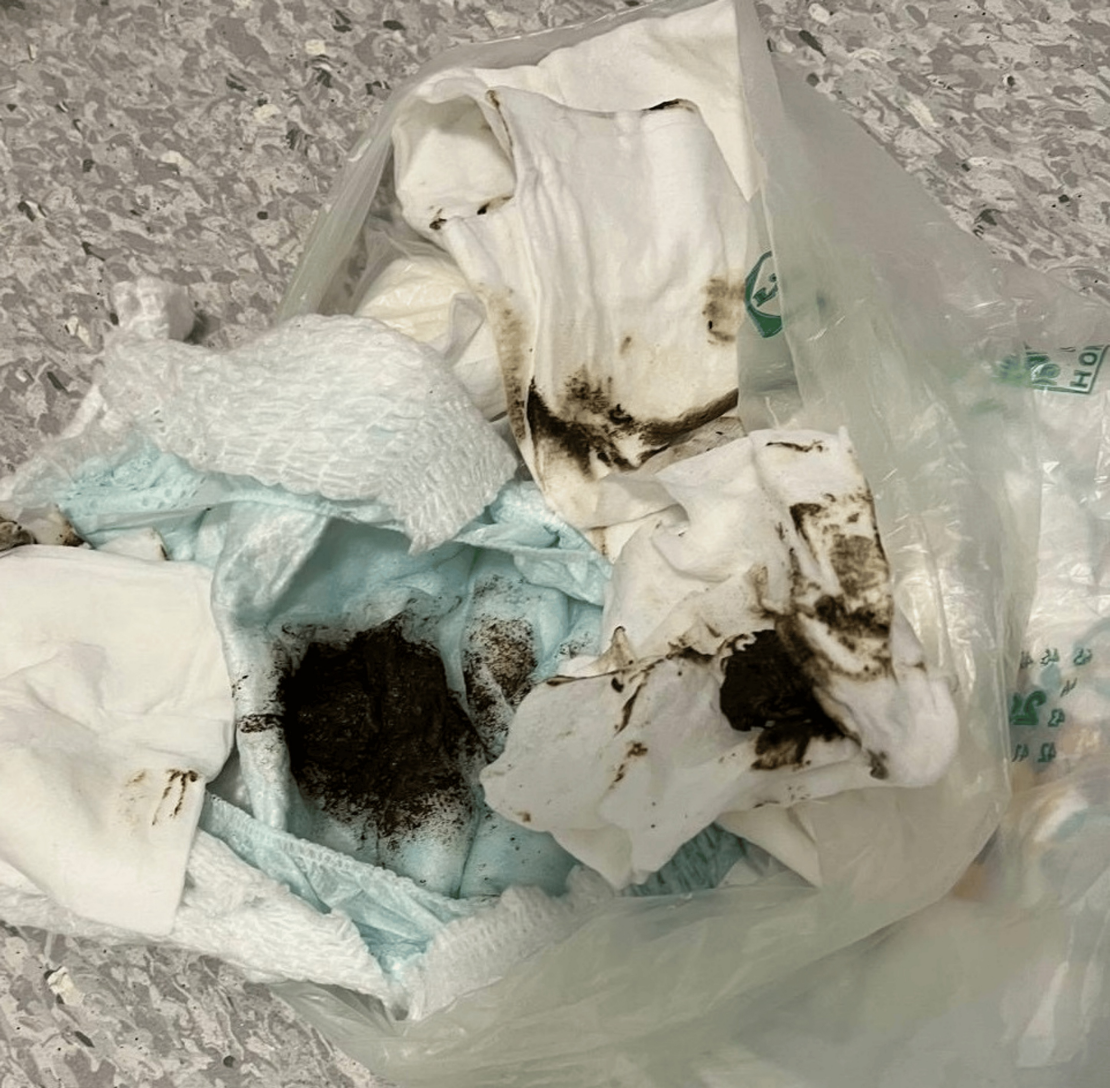
Patient's abnormal stool - appearances in keeping with melaena or black-stained stool

He was brought in by his grandparents, whom he was visiting at the time, with a three-day history of passing dark stool once daily (black semi-formed stool, no comment on offensive smell) (Figure 1) and intermittent complaints of distractable abdominal pain. His initial observations were all normal, except for a heart rate of 158 beats per minute, likely due to his being actively playing in the department and distressed by having his observations taken.

In further discussion of his background, it came to light that he had a history of ingesting non-food items. This included regularly consuming sand and, three days prior, returning home from nursery with a dark blue-stained tongue, which was thought to possibly indicate ink/pigment ingestion. Whilst the change in stool may represent staining from pigment ingestion, there was a high clinical suspicion of melaena.

**Figure 2:**
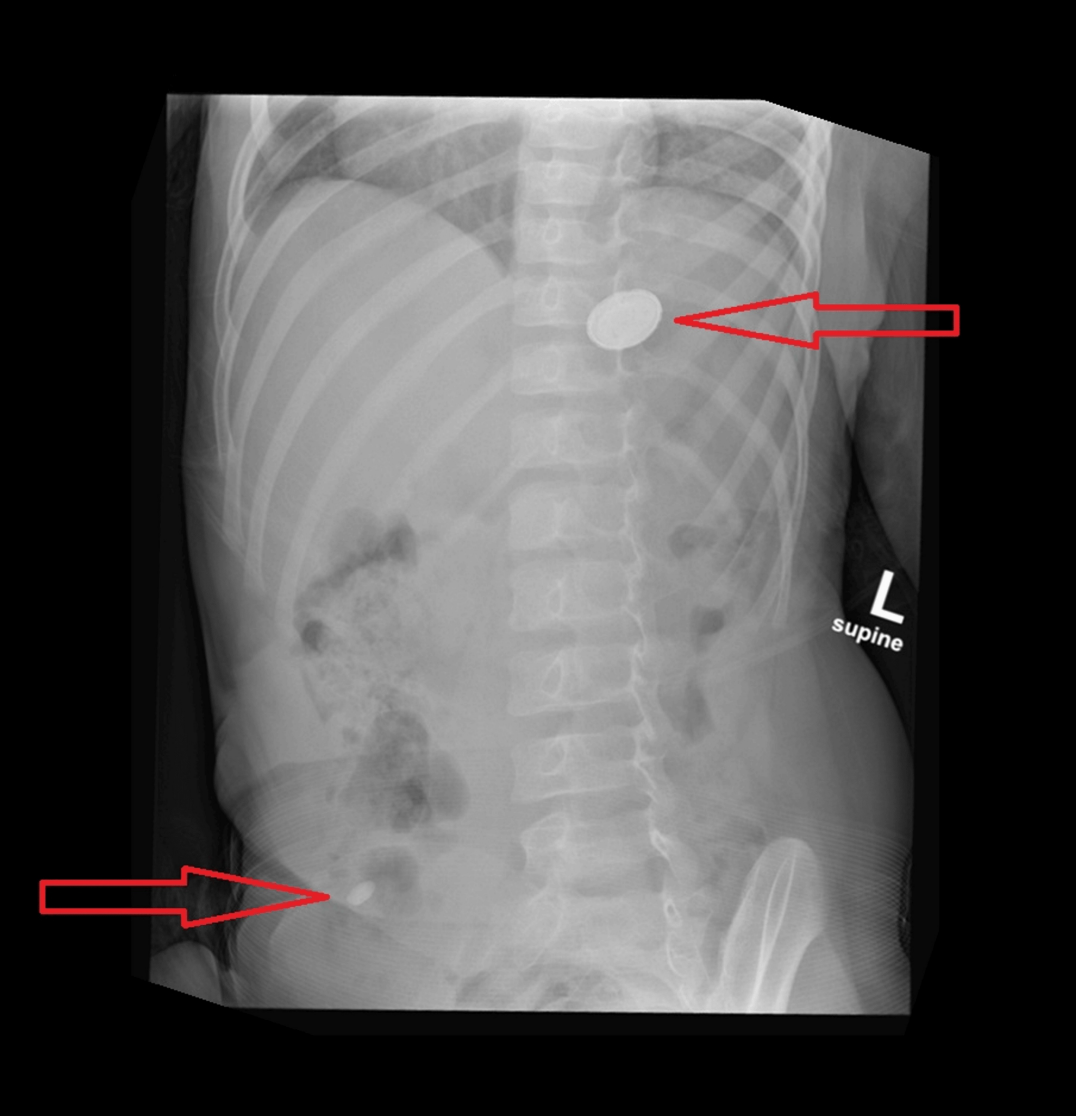
Abdominal X-ray showing two radio-opaque foreign bodies

His initial examination showed no acute abnormalities; his airway, breathing, and circulation were normal, and he had no abdominal tenderness. However, considering his background and stool's appearance, an abdominal X-ray was ordered (Figure 2). This showed a 21.9 mm round radio-opaque object in the stomach with a halo sign indicative of a button battery, alongside a further 9.4 mm radio-opaque object in the right iliac fossa. The abdominal X-ray was of poor quality because it did not include the pelvis, thereby risking a missed diagnosis in the unimaged segment.

Considering the black stool and presence of a button battery, he was discussed with the local paediatric surgical team and transferred to their hospital within 190 minutes of triage. Blood results immediately prior to transfer were normal, apart from mild anaemia, possibly acute (considering the normal mean cell volume and haemoglobin concentrations) (Table 1).

**Table 1:** Pertinent blood results APTT: activated partial thromboplastin time

Test	Result	Normal Range
Haemoglobin	113 g/L	115–140 g/L
Mean cell volume	79.6 fL	73-90 fL
Mean cell haemoglobin concentration	325 g/L	313–352 g/L
Platelet count	292x10^9^/L	150–400x10^9^/L
Prothrombin time	11.1 seconds	11–13.5 seconds
APTT	27.8 seconds	25–5 seconds
APTT ratio	0.95	0.84–1.16
Creatinine	28 μmol/L	23–37 μmol/L
Urea	4.6 mmol/L	2.5–6.5 mmol/L

He underwent endoscopic retrieval within six hours of triage with successful removal of the button battery using rat tooth and then Magill forceps. During endoscopy, there was no significant tissue injury identified, apart from an area of mild erythema at the pylorus. He was observed overnight and discharged home with advice to return if he experiences ongoing symptoms. Since then, he has been doing well, and his melaena has resolved.

## Discussion

This case is notable as it illustrates the need to have a high index of suspicion for unwitnessed ingested foreign objects in those under six years of age, particularly those at higher risk of ingesting foreign objects. Furthermore, it highlights the non-specific symptoms that may present and the difficulty in assessment that some children with communication or behavioural difficulties have.

Button battery ingestion, as in this case report, can present with varied, non-specific symptoms. When not associated with a witnessed ingestion, diagnosis can be significantly challenging. The mechanism of injury from button batteries is via electrochemical damage, potentially leading to necrosis, deep tissue burns and, more seriously, tracheoesophageal or aorto-oesophageal fistula formation [5]. This can cause significant clinical instability and severe symptoms but may also present in a more non-specific manner [6-10].

For example, in this case, the patient attended without any history of vomiting, reduced appetite, distress, drooling, dysphagia, irritability, cough, stridor, or shortness of breath, all of which lowered the index of suspicion for an ingested foreign body. Furthermore, he was unable to explain symptoms or report the ingestion. There was a history of dark stools; photographs taken by family members suggested this was likely melaena. However, there was a risk that this change in his stools could be misattributed to recent pigment ingestion, as indicated by the tongue-staining episode days prior. On balance, considering the communication difficulties, history of foreign body ingestion, melaena, and intermittent abdominal discomfort, the choice to proceed with X-ray imaging was made.

Diagnosis of ingested button batteries is classically through X-ray imaging, ideally with two views. In this case, a single film of the majority of the abdomen was taken, with the key finding being a >20 mm round radiopaque foreign body at the level of the stomach. On inspection, a halo sign was observed, which can help distinguish a coin from a battery [4], although it has been shown in previous studies that in thinner button batteries, a halo sign may not be present [8].

The ESPGHAN guideline was followed under the assumption of a >12-hour delay in diagnosis, and this patient was symptomatic, given the >48-hour history of stool change and intermittent abdominal discomfort. Given that the battery was past the oesophagus, there was no indication to administer honey or sucralfate to reduce the risk of ongoing electrolyte injury [4,9]. If this patient had been asymptomatic, the current guidance would recommend endoscopic removal for high-risk cases (children under five years of age or if the button battery is ≥20 mm) or a wait of 7-14 days, according to the North American Society for Pediatric Gastroenterology, Hepatology, and Nutrition (NASPGHAN) and ESPGHAN guidelines, respectively [4,9]. However, extra caution should be taken with unwitnessed ingestions, as button batteries may transiently lodge in the oesophagus, causing severe erosion. Furthermore, in some cases, gastric necrosis may be asymptomatic in children found to have a gastric button battery [11,12]. Currently, there is a lack of significant evidence to strongly recommend the management of asymptomatic gastric button batteries.

At present, there is no national-level guidance on paediatric button battery ingestion in the UK, with the Royal College of Paediatrics and Child Health (RCPCH), the British Society of Paediatric Gastroenterology, Hepatology and Nutrition (BSPGHAN), and BMJ Best Practice having no guideline (though BSPGHAN references the ESPGHAN position paper) [13-15]. Furthermore, the BMJ Best Practice advice in assessing children with abdominal pain makes no mention of foreign body or button battery ingestion as a differential [16].

Whilst the present study is a single case report and therefore difficult to generalise, the difficulties presented in this case and the lack of national guidance are still of significance. This is in addition to the improved, though still significant, numbers of ingestions among those less than six years of age over the last two decades, between 2004 and 2019, fluctuating between 1,843 and 2,491, peaking in 2007 and lowest in 2019 [1]. Whilst this does show an improvement in the incidence, there is likely further reduction possible.

## Conclusions

In summary, whilst this case report is of a single patient, it highlights the difficulties in assessing high-risk paediatric patients (those under six years of age and those with communication or behavioural difficulties) for foreign body and button battery ingestion. In this case, the only abnormal findings prior to X-ray were photos of black stool, which could have been explained by ink ingestion; intermittent distractable abdominal discomfort on a background of constipation; and a history of non-food item ingestion. The findings of this case show the need for both clinicians and parents to be vigilant of the risk and inconsistent presentation of button battery ingestion in this cohort of patients.

One way to increase clinician awareness of unwitnessed button battery ingestion is to provide national-level guidance on the topic or include it in the differential diagnosis for paediatric abdominal pain guidance. Guardians should be warned about the risk of foreign body ingestion for infants and children at higher risk.

## References

[1] (2025). Button battery ingestions reported to the National Poison Data System (NPDS) and National Battery Ingestion Hotline (NBIH), 1985-2019. https://www.poison.org/battery/stats.

[2] Litovitz T, Whitaker N, Clark L, White NC, Marsolek M (2010). Emerging battery-ingestion hazard: clinical implications. Pediatrics.

[3] Varga Á, Kovács T, Saxena AK (2018). Analysis of complications after button battery ingestion in children. Pediatr Emerg Care.

[4] Mubarak A, Benninga MA, Broekaert I (2021). Diagnosis, management, and prevention of button battery ingestion in childhood: a European Society for Paediatric Gastroenterology Hepatology and Nutrition position paper. J Pediatr Gastroenterol Nutr.

[5] Anfang RR, Jatana KR, Linn RL, Rhoades K, Fry J, Jacobs IN (2019). pH-neutralizing esophageal irrigations as a novel mitigation strategy for button battery injury. Laryngoscope.

[6] Birk M, Bauerfeind P, Deprez PH (2016). Removal of foreign bodies in the upper gastrointestinal tract in adults: European Society of Gastrointestinal Endoscopy (ESGE) clinical guideline. Endoscopy.

[7] Buttazzoni E, Gregori D, Paoli B, Soriani N, Baldas S, Rodriguez H, Lorenzoni G (2015). Symptoms associated with button batteries injuries in children: an epidemiological review. Int J Pediatr Otorhinolaryngol.

[8] Jatana KR, Litovitz T, Reilly JS, Koltai PJ, Rider G, Jacobs IN (2013). Pediatric button battery injuries: 2013 task force update. Int J Pediatr Otorhinolaryngol.

[9] Kramer RE, Lerner DG, Lin T (2015). Management of ingested foreign bodies in children: a clinical report of the NASPGHAN Endoscopy Committee. J Pediatr Gastroenterol Nutr.

[10] Krom H, Visser M, Hulst JM (2018). Serious complications after button battery ingestion in children. Eur J Pediatr.

[11] Lee JH, Lee JH, Shim JO, Lee JH, Eun BL, Yoo KH (2016). Foreign body ingestion in children: should button batteries in the stomach be urgently removed?. Pediatr Gastroenterol Hepatol Nutr.

[12] Ríos G, Rodríguez L, Lucero Y, Miquel I, Arancibia ME, Alliende F (2020). Endoscopic findings associated with button battery ingestion in children: do we need to change the protocol for managing gastric location?. Pediatr Emerg Care.

[13] (2025). Paediatrics and adolescent medicine. https://bestpractice.bmj.com/specialties/22/Paediatrics-and%20adolescent%20medicine.

[14] (2025). Gastroenterology guidelines. https://bspghan.org.uk/gastroenterologyguidelines.

[15] (2025). Clinical guideline directory. https://www.rcpch.ac.uk/resources/clinical-guideline-directory.

[16] (2025). Assessment of abdominal pain in children. https://bestpractice.bmj.com/topics/en-gb/787.

